# Modelling international spread of clade IIb mpox on the Asian continent

**DOI:** 10.2471/BLT.24.291815

**Published:** 2025-06-17

**Authors:** Toshiaki R Asakura, Sung-mok Jung, Hiroaki Murayama, Cyrus Ghaznavi, Haruka Sakamoto, Ayaka Teshima, Fuminari Miura, Akira Endo

**Affiliations:** a Department of Infectious Disease Epidemiology and Dynamics, London School of Hygiene & Tropical Medicine, London, England.; b Carolina Population Center, University of North Carolina at Chapel Hill, Chapel Hill, United States of America (USA).; c School of Medicine, International University of Health and Welfare, Narita, Japan.; d Department of Medicine, University of California San Francisco, San Francisco, USA.; e Graduate School of Public Health, St. Luke’s International University, Tokyo, Japan.; f School of Medicine and Health Sciences, Universitat de Barcelona, Barcelona, Spain.; g Centre for Infectious Disease Control, National Institute for Public Health and the Environment, Bilthoven, Kingdom of the Netherlands.; h Saw Swee Hock School of Public Health, National University of Singapore, 12 Science Drive 2, #10-01, 117549, Singapore.

## Abstract

**Objective:**

To understand and simulate international spread of the disease mpox, considering variations in sexual activity levels and international travel among men who have sex with men.

**Methods:**

We developed a mathematical model that considers differing sexual networks and the volume of international travel among men who have sex with men, calibrated to disease incidence data in Japan. We then used our model to simulate the potential international spread of mpox across 42 countries and territories on the Asian continent, assuming Japan as the origin of spread.

**Findings:**

Our simulations identified countries and territories at a high risk of mpox introduction, many being low- and middle-income countries and territories in the Western Pacific and South-East Asia regions. We found that the simulated risk of importation gradually shifted over time from the Western Pacific to the South-East Asia region, and later to the Eastern Mediterranean and European regions. This simulated pattern broadly aligns with actual mpox spread patterns observed between 2023 and 2024.

**Conclusion:**

Our multicountry model for mpox outbreaks can help project the possible trajectory of mpox spread across countries and territories on the Asian continent. Our findings warrant global efforts to contain mpox outbreaks, particularly support for low- and middle-income countries and territories which are at higher risk of introduction, so that the risk of continued spread across the Asian continent and beyond is reduced.

## Résumé

**Objectif:**

Comprendre et simuler la propagation internationale de la variole simienne (mpox), en tenant compte des variations des niveaux d’activité sexuelle et des voyages internationaux parmi les hommes ayant des relations sexuelles avec d’autres hommes.

**Méthodes:**

Nous avons mis au point un modèle mathématique qui prend en compte les différents réseaux sexuels et le volume des voyages internationaux parmi les hommes ayant des relations sexuelles avec d’autres hommes, calibré sur les données d’incidence de la maladie au Japon. Nous avons ensuite appliqué notre modèle pour simuler la propagation internationale potentielle de la mpox dans 42 pays et territoires du continent asiatique, en supposant que le Japon soit à l’origine de la propagation.

**Résultats:**

Nos simulations ont permis d’identifier les pays et territoires présentant un risque élevé d’introduction de la mpox, dont un grand nombre sont des pays et territoires à revenu faible ou intermédiaire des régions du Pacifique occidental et d’Asie du Sud-Est. Nous avons constaté que le risque simulé d’importation s’est progressivement déplacé, au fil du temps, du Pacifique occidental vers la région de l’Asie du Sud-Est, puis vers les régions de la Méditerranée orientale et de l’Europe. Ce modèle simulé correspond généralement aux modèles de propagation de la mpox observés entre 2023 et 2024.

**Conclusion:**

Notre modèle multinational pour les épidémies de mpox permet de projeter la trajectoire possible de la propagation de cette maladie dans les pays et territoires du continent asiatique. Nos résultats justifient les efforts déployés à l’échelle mondiale pour contenir les épidémies de mpox, en particulier le soutien aux pays et territoires à revenu faible et intermédiaire qui présentent un risque d’introduction plus élevé, afin de réduire le risque de propagation continue sur le continent asiatique et au-delà.

## Resumen

**Objetivo:**

Comprender y simular la propagación internacional de la enfermedad mpox, considerando las variaciones en los niveles de actividad sexual y en los desplazamientos internacionales entre hombres que tienen relaciones sexuales con hombres.

**Métodos:**

Se desarrolló un modelo matemático que incorpora redes sexuales diferenciadas y volúmenes de viajes internacionales entre hombres que tienen relaciones sexuales con hombres, calibrado con datos de incidencia de la enfermedad en Japón. Posteriormente, se utilizó este modelo para simular la posible propagación internacional de la mpox en 42 países y territorios del continente asiático, considerando a Japón como punto de origen de la propagación.

**Resultados:**

Las simulaciones identificaron países y territorios con alto riesgo de introducción de la mpox, muchos de ellos ubicados en países y territorios de ingresos bajos y medianos de las Regiones del Pacífico Occidental y Asia Sudoriental. Se observó que el riesgo simulado de importación se desplazaba gradualmente desde el Pacífico Occidental hacia Asia Sudoriental, y posteriormente hacia las Regiones del Mediterráneo Oriental y Europa. Este patrón simulado se corresponde, en términos generales, con la propagación real de la mpox observada entre 2023 y 2024.

**Conclusión:**

El modelo multinacional desarrollado para los brotes de mpox permite proyectar la posible trayectoria de su propagación en países y territorios del continente asiático. Los resultados obtenidos justifican los esfuerzos globales para contener los brotes de mpox, con especial atención al apoyo a los países y territorios de ingresos bajos y medianos, que presentan mayor riesgo de introducción, a fin de reducir el riesgo de propagación continua en Asia y más allá.

## ملخص

الغرض فهم ومحاكاة الانتشار الدولي لفيروس mpox، مع الوضع في الاعتبار الاختلافات في مستويات النشاط الجنسي، والسفر الدولي، بين الرجال الذين يمارسون الجنس مع رجال.

الطريقة قمنا بتطوير نموذجًا رياضيًا يأخذ في الاعتبار الشبكات الجنسية المختلفة، وحجم السفر الدولي بين الرجال الذين يمارسون الجنس مع رجال، بعد معايرته ببيانات معدل الإصابة بالمرض في اليابان. ثم استخدمنا نموذجنا لمحاكاة الانتشار الدولي المحتمل لفيروس mpox عبر 42 دولة ومنطقة في قارة آسيا، بافتراض أن اليابان هي مصدر الانتشار.

النتائج حددت عمليات المحاكاة الدول والمناطق المعرضة لخطر كبير لانتشار فيروس mpox، والعديد منها دول وأقاليم منخفضة ومتوسطة الدخل في منطقتي غرب المحيط الهادئ وجنوب شرق آسيا. ووجدنا أن خطر الاستيراد المُحاكي قد تحول تدريجيًا بمرور الوقت من غرب المحيط الهادئ إلى منطقة جنوب شرق آسيا، ولاحقًا إلى منطقتي شرق البحر الأبيض المتوسط وأوروبا. يتوافق هذا النمط المُحاكي بشكل عام مع أنماط انتشار فيروس mpox الفعلية المُلاحظة بين عامي 2023 و2024.

الاستنتاج يُمكن للنموذج متعدد الدول لدينا لانتشار فيروس mpox أن يُساعد في توقع المسار المُحتمل لانتشار فيروس mpox عبر دول وأقاليم قارة آسيا. وتُبرر نتائجنا بذل جهود عالمية لاحتواء انتشار فيروس mpox، لا سيما دعم الدول والأقاليم منخفضة ومتوسطة الدخل الأكثر عُرضةً لخطر تفشيه، وذلك للحد من خطر استمرار انتشاره عبر قارة آسيا وخارجها.

## 摘要

**目的:**

通过考虑男男性行为者在性行为频率和国际旅行方面的变化，了解并模拟猴痘疾病的国际传播情况。

**方法:**

我们构建了一个数学模型，该模型将不同的性网络以及男男性行为者的国际旅行次数纳入了考量范围，并基于日本的疾病发病率数据进行校准。然后，在假设日本是病毒传播起源地的前提下，我们运用该模型模拟了猴痘病毒在亚洲大陆 42 个国家和地区的潜在国际传播情况。

**结果:**

我们经过模拟分析确定了猴痘病毒传入风险较高的国家和地区，其中很多都是西太平洋和东南亚区域的中低收入国家和地区。我们经过模拟分析发现，随着时间的推移，猴痘病毒可能会逐渐从西太平洋区域传入东南亚区域，随后又进而传入东地中海和欧洲区域。据我们观察，该模拟传播模式与 2023 年至 2024 年期间的实际猴痘病毒传播模式大致相符。

**结论:**

我们针对猴痘疫情构建的多国模型可能有助于预测亚洲大陆各个国家和地区猴痘病毒的可能传播轨迹。我们的分析结果表明，全球各个国家和地区必须联合行动以防止猴痘疫情蔓延，特别是为传入风险更高的中低收入国家和地区提供援助，从而降低猴痘病毒在整个亚洲大陆以及其他地区持续传播的风险。

## Резюме

**Цель:**

Получить представление о международном распространении такого заболевания, как оспа обезьян, и составить модель этого процесса, учитывая различия в уровнях сексуальной активности и международных поездок среди мужчин, практикующих секс с представителями того же пола.

**Методы:**

Авторы разработали математическую модель, в которой учитывается наличие различных сексуальных сетей общения и объем международных путешествий среди мужчин, практикующих секс с представителями того же пола, и откалибровали ее по данным о заболеваемости оспой обезьян в Японии. Затем эту модель использовали для моделирования потенциального распространения оспы обезьян в 42 странах и регионах Азии, приняв Японию за начальную точку.

**Результаты:**

Моделирование показало, что высокий риск интродукции оспы обезьян характерен для многих стран и территорий с низким и средним уровнем дохода, расположенных в западной части Тихоокеанского региона и в Юго-Восточной Азии. Было обнаружено, что смоделированный риск завоза заболевания постепенно сместился из западной части Тихоокеанского региона в Юго-Восточную Азию, а затем в восточную часть Средиземноморья и в Европу. Смоделированный паттерн распространения в значительной мере совпадает с фактическим паттерном распространения оспы обезьян, который наблюдался в период с 2023 по 2024 годы.

**Вывод:**

Модель для анализа вспышек оспы обезьян в разных странах, представленная авторами, может помочь наметить возможную траекторию распространения оспы обезьян по странам и регионам Азиатского континента. Результаты исследования говорят о необходимости международных усилий по локализации вспышек оспы обезьян, в частности о том, что необходимо оказывать поддержку странам и регионам с низким и средним уровнем дохода, которые находятся в зоне высокого риска интродукции инфекции. Это позволит снизить риск дальнейшего распространения заболевания по Азиатскому континенту и за его пределы.

## Introduction

The global spread of clade IIb mpox outbreaks among men who have sex with men expanded rapidly across numerous countries in the Region of the Americas and the European Region within months of its emergence in May 2022.^1^ After reaching around 40 000 cases worldwide, cases started to decline by late 2022.^1^ Subsequently, local transmission largely subsided in those regions, with only some sporadic and limited resurgences occurring from 2023 onwards.^2^ Nevertheless, as of April 2025, clade IIb continues to drive a prolonged global outbreak primarily affecting men who have sex with men, with cases reported worldwide.^3^

Although annual case numbers outside the African Region (primarily representing clade IIb cases) have declined since 2022 (when 84 017 cases were recorded), they remain substantial, with 9198 cases reported in 2023 and 10 728 cases in 2024. Mpox outbreaks have continued into 2025, with 1662 cases reported outside the African Region between January and March.^4^

Until the end of 2022, the Asian continent was the least affected region globally, with only a small number of imported cases and limited onward transmission^5^. In 2023, however, the pattern shifted following the onset of a local outbreak in Japan in January – with only three affected people having a history of international travel.^6^ During 2023 to 2024, several countries in the Western Pacific and South-East Asia regions experienced sustained mpox transmission. China reported the largest epidemic in the region with 2806 cases by the end of 2024,^5^^,^^7^ followed by Thailand with 853 cases. Phylogenetic analyses^8^ showed that most viral samples from affected countries and territories were distinct from the lineages that circulated in other regions in 2022 and were linked to samples from Japan. These findings suggest that mpox epidemics on the Asian continent since 2023 could be characterized as a divergent regional outbreak.

Despite the World Health Organization lifting its first declaration of mpox as a public health emergency of international concern on 11 May 2023,^9^ international spread remains a growing threat to the Asian continent. Many Asian countries and territories, most of which are in low- and middle-income countries with limited access to mpox vaccines, have large, susceptible populations at risk.^6^ These unique conditions warrant quantitative risk assessments for regional outbreaks of mpox.

Mathematical models used to study the international spread of emerging infectious diseases have conventionally combined data on local transmission dynamics with data on global import, characterized by international travel volume.^10^^,^^11^ These models have been successful in understanding and predicting the spatiotemporal patterns of global spread of respiratory pathogens such as the severe acute respiratory syndrome coronavirus 2^12^^–^^14^ and the influenza virus.^10^^,^^15^ However, such models typically assume homogenous mixing among populations; this limits how applicable they are to the global outbreak of clade IIb mpox, where highly heterogeneous sexual networks among men who have sex with men play a substantial role in shaping transmission dynamics.^16^^–^^20^ Within such networks, individuals with higher numbers of sexual partners are more likely to be infected and to acquire immunity in the early phase of an outbreak,^21^^,^^22^ while those with fewer sexual partners remain susceptible. Since we assume the number of sexual partners is proportional to the reproduction number, the risk of onward transmission per infector declines as the outbreak progresses. A previous global modelling study highlighted the importance of accounting for this selective transmission when examining the international spread of clade IIb mpox in 2022.^23^ As mpox outbreaks on the Asian continent from 2023 onward have continued to primarily affect men who have sex with men, it is essential to incorporate this depletion-of-susceptibles due to sexual network heterogeneity when analysing patterns of international spread.

## Methods

In this study, we developed a stochastic model of mpox transmission over sexual contact networks among men who have sex with men, in which we incorporated travel volume to simulate mpox spread patterns across borders on the Asian continent. The study was initially designed to evaluate the prospective risk of mpox importation across Asian countries and territories starting from July 2023, based on data available at time of the analysis.^24^ Specifically, we fitted our model to mpox incidence data in Japan and confirmed that the simulated international spread generally aligned with countries reporting importations as of July 2023. Subsequently, we extended our simulations to project future mpox international spread patterns across the Asian continent.

In this paper, we maintain the original scope and data timeframe of our simulation and present a retrospective analysis by comparing our projections with the actual evolution of the outbreak since the time of the initial analysis.

### Data source

We collected public mpox incidence data in Japan from the Ministry of Health, Labour and Welfare,^25^ covering the period from 25 July 2022 to 7 July 2023.

To model sexual mixing patterns among men who have sex with men, we used the sexual partnership distribution estimated from self-reported sexual partner counts over a 4-week period in the United Kingdom of Great Britain and Northern Ireland’s National Survey of Sexual Attitudes and Lifestyles data sets, since comparable data sets in Asia were not available.^26^^,^^27^ We also used the sexual partnership distribution over 1 year estimated from this survey as part of our sensitivity analysis (online repository).^16^^,^^28^

Data on international travel volume in 2019 were sourced from the World Tourism Organization.^29^ Countries and territories included in our study were those classified as the Asia region according to the United Nations Geoscheme, spanning across (but not inclusive of) the World Health Organization’s South-East Asia, European, Eastern Mediterranean and Western Pacific regions.^30^ Afghanistan, the Democratic People's Republic of Korea, Iraq, Pakistan, Turkmenistan and Yemen were excluded due to missing data on international travel volume.

To qualitatively validate our international projections of mpox, we compiled the dates of the first reported mpox incidence since 2023 across Asian countries and territories from multiple sources. We also obtained the cumulative mpox case counts from the Our World in Data website.^5^

To assess the plausibility of our assumption that sexual partnership distributions on the Asian continent resemble those in the United Kingdom survey data set, we analysed data from the National Inventory of Japanese Sexual Behaviour (online repository).^28^^,^^31^ Except for the National Inventory of Japanese Sexual Behaviour study data, which is subject to ethics-based restrictions on data sharing (details are available in the original study),^31^ all data used in this study are publicly available.

### Model of spread

We developed a stochastic meta-population susceptible-exposed-infected-recovered model to represent populations of men who have sex with men across the 42 study countries and territories. Due to limited availability and substantial uncertainty in population size estimates for men who have sex with men among these countries and territories, we assumed that men who have sex with men account for 1% of the total population (i.e. 2% of the male population) in every country or territory considered.^32^ The rationale for our assumptions are detailed in the online repository.^28^

To capture heterogeneity in sexual behaviours driving mpox transmission,^17^ we stratified each country or territory’s population of men who have sex with men into 50 groups, according to different levels of sexual activity. These levels – defined by the number of sexual partners over mpox’s assumed 10-day infectious period^33^ – were assumed to be linearly correlated with both infection risk and onward transmission. 

We did not consider the effect of vaccination against mpox in our analysis as Japan’s only approved mpox vaccine as of 2023 was administered exclusively in clinical trial settings and not widely available for prevention.^34^

Mpox importations between countries and territories were modelled using 2019 travel volume data from the United Nations World Tourism Organization.^29^ We assumed that the importation risk between any two locations was proportional to total travel volume – accounting for both inbound and outbound travellers.

We simulated the international spread of mpox across countries and territories using a Bayesian data assimilation framework.^35^^,^^36^ Specifically, we calibrated our model to the reported mpox incidence in Japan between 16 January 2023 (the date of medical attendance for the first case) and 26 June 2023, using approximated Bayesian computation^36^ to yield the posterior samples (parameter values consistent with the data) of the sexually-associated secondary attack risk (that is, the transmission risk per sexual partnership) for mpox. To align with the observed local transmission in the Republic of Korea by the end of the fitting period (26 June 2023), we also conditioned (constrained) the posterior samples on the early introduction into the Republic of Korea by only accepting a subset of posterior samples; i.e. the model was required to have 10 or more cases in the Republic of Korea by 26 June 2023 for samples to be accepted. We then simulated mpox transmission from 16 January 2023 onwards in Japan and other countries and territories on the Asian continent, incorporating international travel volume data.^29^ For simplicity, we assumed that Japan served as the source of international mpox spread on the Asian continent from 2023, and that importations from outside the Asian continent were negligible. Further methodological details can be found in the online repository.^28^

## Results

### Simulated importation probabilities

Calibration of our model using mpox incidence data in Japan from 16 January 2023 to 26 June 2023 gave a posterior median estimate for the sexually-associated secondary attack risk of 72% (95% credible interval: 47–93). This estimate aligns closely with findings from a contact tracing study in Belgium conducted during the 2022 outbreak which reported a secondary attack risk of 66% (12 positive cases among 18 contacts).^37^

**Fig. 1:**
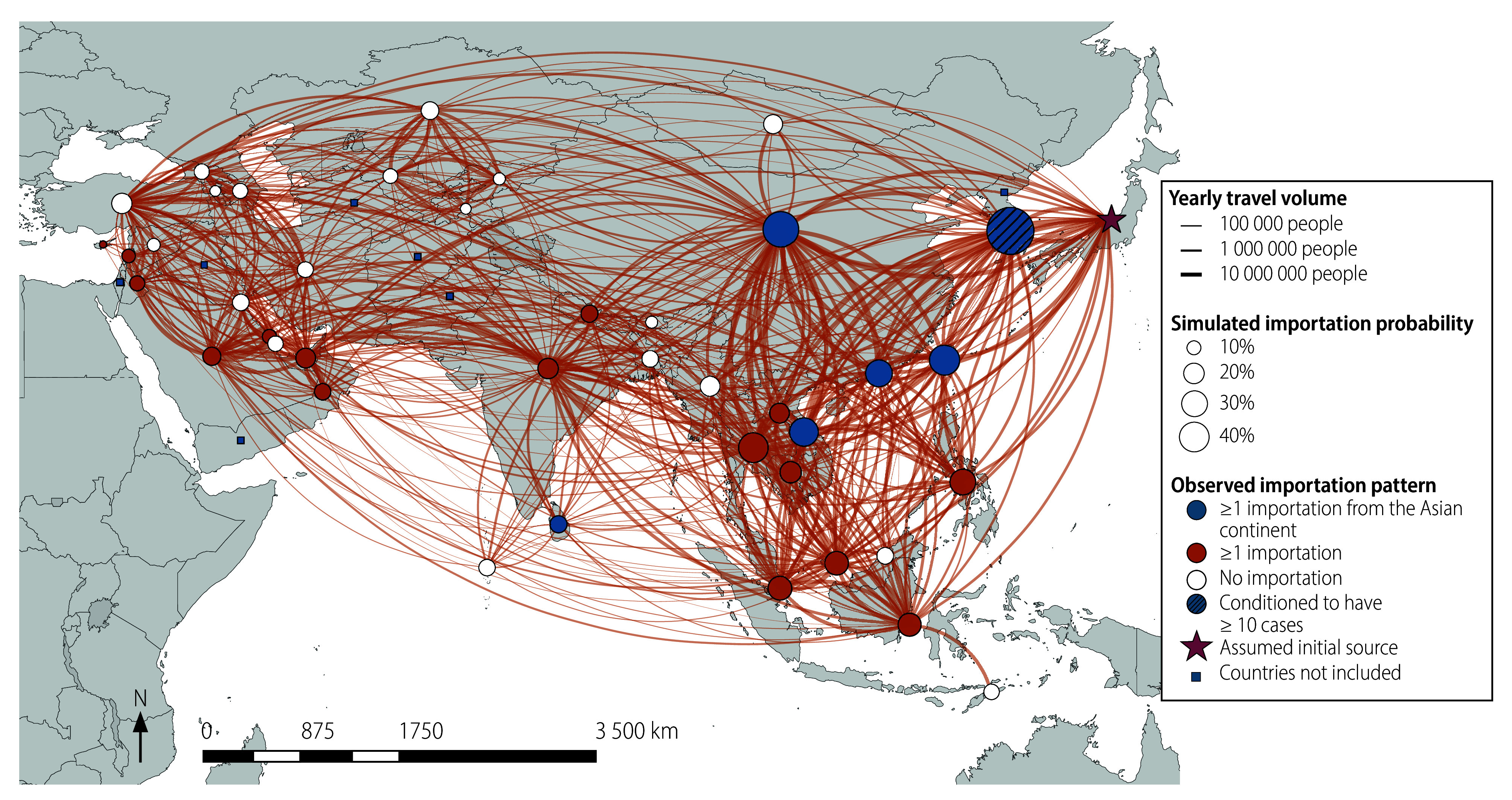
Simulated mpox importation probabilities since 2023, Asian continent

Notes: the node size represents the simulated importation probability for each country or territory under the condition of having ≥ 10 mpox cases in Republic of Korea (hatched node). The red star represents the assumed initial source of spread from Japan. Links for travel volume of less than 10 000 persons per year were omitted from the figure.

Using these posterior samples, we computed the simulated importation probabilities for each country or territory, which we defined as the proportion of simulations in which at least one case was imported (Fig. 1). Countries and territories with the highest simulated importation probabilities were mostly concentrated in the South-East Asia and Western Pacific regions. Of note, six of the top 10 countries and territories with the highest importation probabilities (mainland China, Indonesia, Malaysia, Philippines, Thailand and Viet Nam) were classed as low- and middle-income countries. Similarly, 13 of the top 20 were low- and middle-income countries or territories. We also found that many of these countries and territories later reported mpox introductions in 2023–2024, consistent with our model projections (online repository).^28^

### Simulated mpox spread

**Fig. 2:**
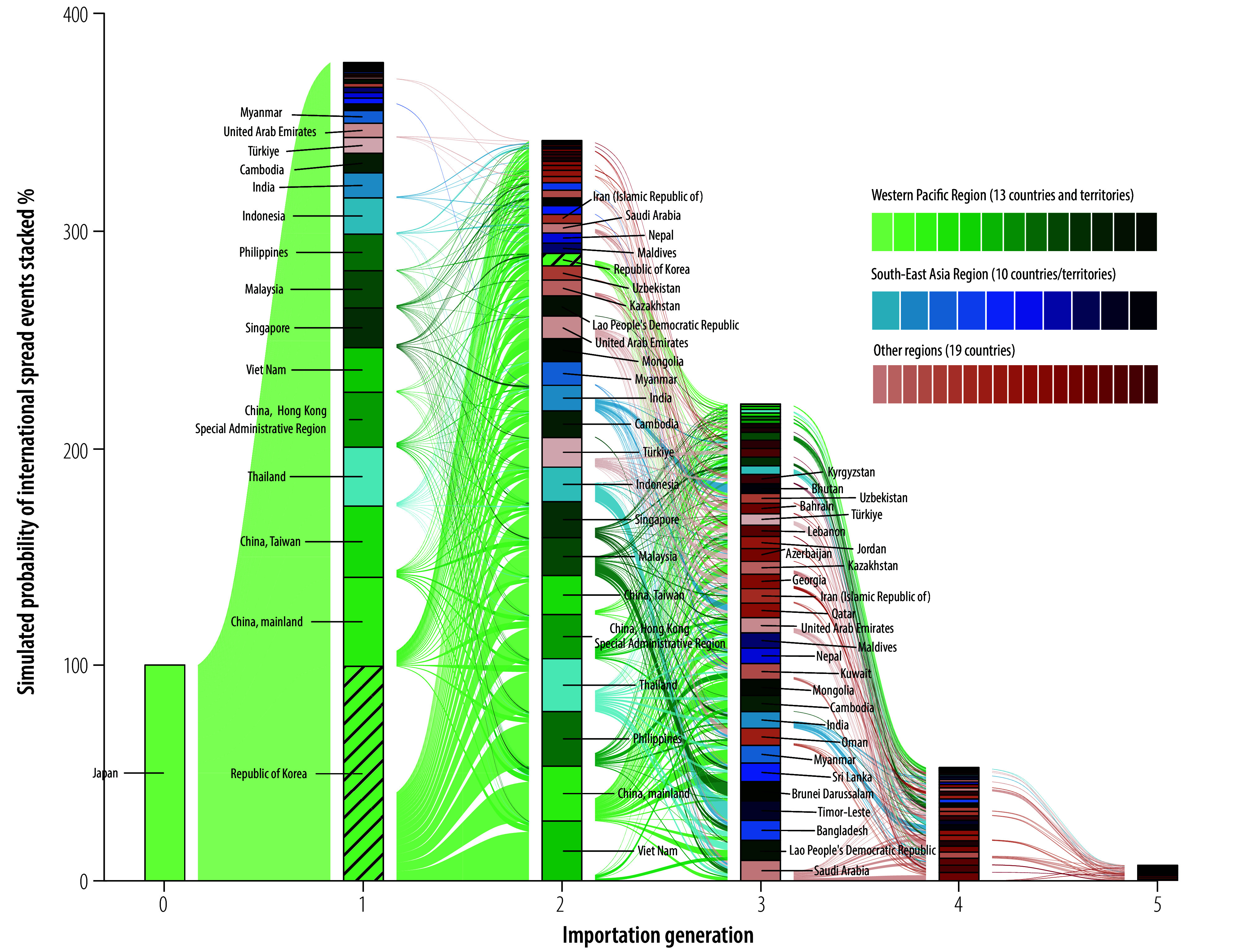
International mpox spread patterns since 2023, Asian continent

Notes: each column represents the generation of international spread. The height of each box denotes the simulated probability of international spread events for each generation conditional to ≥ 10 mpox cases in Republic of Korea (hatched boxes). The width of the outflow from the right-hand side of each box denotes the relative frequency of exportation. Other regions include the European and Eastern Mediterranean regions.

We visualized the projected international spread of mpox on the Asian continent using an alluvial diagram (Fig. 2). We defined an international spread event as the first importation of an mpox case between a specific importation-exportation country or territory pair since 2023. In the figure, the boxes and connecting paths represent the simulated probabilities of these events and their generational order, assuming Japan as the initial source of spread. Note that as per our methods, these simulations were conditioned on ≥ 10 cases in the Republic of Korea. Most first-generation importations (i.e. direct importations from Japan) were to countries and territories in the Western Pacific Region, while second-generation spread predominantly involved countries in the South-East Asia Region. From the third-generation onward, the proportion of importations involving the European and Eastern Mediterranean regions increased, albeit with relatively low simulated importation probabilities.

## Discussion

Our multicountry mpox transmission model, which considered heterogeneous sexual contact networks and international travels, projected possible patterns of international mpox spread across the Asian continent. Simulations assumed the mpox outbreak originated in Japan, which experienced the earliest sustained local outbreak among countries and territories on the Asian continent in 2023.

Our results identified countries and territories at high risk of importation, many of which did report importations in 2023–2024, including several low- and middle-income settings in the South-East Asia and Western Pacific regions. This importation risk is of particular concern due to the large population sizes and limited access to mpox vaccines in these countries and territories. A large outbreak in China during the summer of 2023,^5^ followed by reported cases in neighbouring countries and territories,^38^ underscored this regional concern.

Although the absolute simulated importation risks should be interpreted with caution – owing to stochastic variability in outbreak sizes between simulation runs (online repository)^28^ – the relative importation patterns remained robust, including in our sensitivity analyses (online repository).^28^ We therefore believe that, despite caveats, our simulation would help anticipate the possible future trajectory of mpox spread across the Asia continent. Notably, new data on mpox introductions reported from 2023 to 2024 – initially from the Western Pacific Region, followed by the South-East Asia Region – have so far aligned with our simulated projections. Whether sustained transmission will extend to the Eastern Mediterranean and European Regions warrants close attention from the global health community given the risk of global resurgence of clade IIb mpox.

The global outbreak of clade IIb mpox in 2022 primarily affected the Region of the Americas and the European Region, where the imminent public health threat has since diminished,^9^ likely due to immunity being established as a result of natural infection or vaccination.^17^ However, multiple events suggest potential risk of reemergence, i.e. new cases in areas where the epidemic previously appeared to have ceased,^39^^,^^40^ and reports of breakthrough infections and reinfections.^41^

Even if the transmission potential of mpox has decreased due to accumulated immunity in a population, multiple factors including waning immunity, the replenishment of susceptible individuals among the sexually active group (e.g. due to sexual debut or behavioural changes) or the emergence of immune escape variants could still drive reemergence in the long term. Indeed, several countries including Australia, Brazil and Canada experienced a major mpox resurgence in 2024; Australia, for instance, recorded its highest-ever monthly case count of 302, five times the peak monthly count recorded during the first wave in 2022.^4^ Continued circulation of mpox in the Western Pacific Region is therefore of concern not only for this region, but also for other regions – including those that were affected in 2022. Global support for control efforts in low- and middle-income countries in the South-East Asia and Western Pacific regions, as well as other regions at increased risk, remains essential.

Our study has several limitations. First, we assumed uniform sexual partner distributions and a fixed per-capita size for the population of men who have sex with men across the Asian continent, but this may not fully reflect heterogeneity across diverse socio-cultural backgrounds. As existing data sets on partnerships of men who have sex with men in the included countries and territories did not contain the full distribution to inform our model, we used the United Kingdom survey data set as a proxy. Nevertheless (after adjusting the reporting time window for partners), we found that existing data sets from mainland China; China, Taiwan; Japan; Malaysia; Singapore and other countries and territories in South-East Asia and Western Pacific regions^31^^,^^42^^,^^43^ generally suggested similarity in partner distributions – including in settings where homosexuality is criminalized^44^ (online repository).^28^ Second, in our model we did not incorporate pre-existing immunity (infection- or vaccine-derived) or behavioural changes, both of which have been discussed in previous mpox modelling studies from high-income countries in the Region of the America and the European Region.^18^^–^^20^^,^^45^^,^^46^ Given low case counts and limited vaccine roll-out in most Asian countries and territories by 2023, we assume prior immunity was likely to be minimal. It also remains unclear whether there were measurable behavioural changes in response to the mpox outbreak on the Asian continent in 2023, particularly after WHO’s first declaration of the mpox outbreak as a public health emergency of international concern was lifted.^9^ Predicting future behavioural responses was out of scope for our model, which was originally designed for forward-looking mpox outbreak projections as of July 2023.^24^ However, we partially accounted for possible behavioural changes in our sensitivity analysis (online repository).^28^ Third, we used 2019 travel volume data from the United Nations World Tourism Organization as a proxy for 2023 data (which was unavailable at the time of our analysis). Although travel volumes in 2023 may have differed due to lingering impact of the coronavirus disease 2019 pandemic (online repository),^28^ our sensitivity analysis incorporating additional flight passenger data^47^ suggested the use of 2019 travel volume data instead of 2023 data had limited impact on results (online repository).^28^ Finally, our model restricted mpox spread to within the Asian continent; some importations – particularly, into the East Mediterranean Region – may have originated from outside the Asian continent. Early importations into this region that occurred before our simulation period (online repository)^28^ may reflect introductions from Europe.

In summary, our simulation highlights ongoing risks of mpox importation across the Asian continent, particularly among low- and middle-income countries with large, susceptible populations and limited access to vaccines. Although the overall case trend in Asian countries and territories has shown a gradual decline since late 2023, the most recent data suggests persisting transmission in the region,^3^ keeping susceptible populations at further risk of importation and local spread. The importation pathway and risk patterns identified in our study offer a useful basis for assessing outbreak response strategies. Despite intensifying global concern about the more recent emergence of mpox clade Ib, clade IIb remains the dominant public health threat on the Asian continent as of April 2025. Strengthened surveillance, concerted global efforts, and vaccine equity remain essential to containing future mpox outbreaks.
